# Optimization of a Cytochrome-P450-Monooxygenase-1A-Mediated EROD Assay in the Cape Hake Species *Merluccius capensis* and *Merluccius paradoxus* (Pisces)

**DOI:** 10.4061/2011/108395

**Published:** 2011-11-22

**Authors:** Louise De Almeida, William Froneman, Brett Pletschke

**Affiliations:** ^1^Department of Biochemistry, Microbiology and Biotechnology, Rhodes University, Grahamstown 6140, South Africa; ^2^Department of Zoology and Entomology, Rhodes University, Grahamstown 6140, South Africa

## Abstract

Cytochrome P450 monooxygenase 1A (CYP1A) is induced by several planar toxic compounds, for example, polychlorinated biphenyls (PCBs) and the induction of this protein is often measured in terms of CYP1A-mediated 7-ethoxyresorufin-*O*-deethylase (EROD) activity. This study was aimed at developing this assay in the Cape hake species *Merluccius capensis* and *Merluccius paradoxus* (considered one stock). Microsomal fractions were obtained from frozen fish liver samples by differential centrifugation. Fluorimetric and spectrophotometric analysis of the EROD assay resulted in the spectrophotometric (at 572 nm) detection method being selected, as this method resulted in a lower degree of variability and demonstrated higher reproducibility. The activity in the EROD assay was enhanced in the presence of NADPH, and the addition of dicumarol (phase II enzyme inhibitor) to the reaction mixtures prevented the underestimation of this assay by the inhibition of DT-diaphorase. In summary, an EROD assay was established for use in Cape hake species.

## 1. Introduction

In recent years the increased production and release of organic trace pollutants, for example, herbicides, metals, polychlorinated biphenyls, alkylphenols, insecticides and industrial effluent mixtures into the marine environment have increased concern and awareness into the bioaccumulation, bioconcentration, and biomagnification of these pollutants in marine organisms [1–3]. The production and release of these pollutants have been directly linked to the reduction in successful reproduction and increased mortality in several fish species [4–6]. In many fish populations, the pollutant toxic effects are only evident after an extended period of time. As a result, research into early warning systems in the form of biomarkers for various pollutants has received increased attention [7, 8]. A biomarker is defined as a biological response (molecular, physiological, or behavioral) which can be traced back to the exposure or the toxic effect of environmental pollutants. These effects can be measured in body fluids, cells, or tissues [9]. The use of biomarkers has several advantages over the use of analytical chemistry for the detection of pollutants in the aquatic environment. Biomarkers do not require the use of different chemicals at varying concentrations and thus do not introduce “foreign” chemicals into the environment. Also, the use of analytical techniques can often be very expensive and requires specialized training [10].

Fish models have played a significant role in toxicological studies used to assess the state of aquatic environments [11, 12]. The use of fish models poses several advantages because these organisms are in constant contact with the environment and thus may be directly affected by a variety of chemicals [13]. These models have also proven to be cost effective [11, 12].

The aim of the present study was to optimize the cytochrome P450 monooxygenase 1A (CYP1A) 7-ethoxyresorufin deethylation (EROD) reaction for Cape hake. The superfamily of cytochrome P450s are heme containing proteins which regulate the metabolism (phase I metabolism) of several xenobiotic and endogenous compounds [14]. CYP1A belongs to a subfamily of the P450 superfamily which is found predominantly in the liver but has also been found in the kidneys, gill tissue, and endoplasmic reticula of fish [15, 16]. CYP1As are induced by polychlorinated dibenzo-*p*-dioxins (PCDDs), polychlorinated dibenzofurans (PCDFs), and polychlorinated biphenyls (PCBs) [17–19]. CYP1A genes are activated by pollutant compounds via high affinity competitive binding to the aryl hydrocarbon receptor [18]. CYP1A is one of the most widely used biomarkers for the detection of chemical contamination in the aquatic environment because CYP1A activity is highly susceptible to the effects of toxic compounds [20–22]. 

The induction of CYP1A is most commonly measured in terms of EROD activity, as this indirect strategy has proven to be cost-effective and sensitive even in a complex mixture of compounds [23]. The EROD assay demonstrates the effect of the uptake of toxic planar compounds in fish, whether the presence of these agents has been analytically detected or not [24–26].

*Merluccius capensis *and *Merluccius paradoxus *(Cape hake) were used in this study as the model species for the development of the EROD assay. Cape hake belong to the family Gadidae and coinhabit Namibian and South African waters [27]. Their geographical distribution is associated with the Benguela Current system (17°30′S–29°30′S) [28]. *M. capensis *and *M. paradoxus *are morphologically similar and the differences that separate them, for example, differences in the number of vertebrae are very minor [29, 30]. In this study the two species were, therefore, considered one stock and no species differentiation was conducted. *M. capensis *occurs predominantly off the coast of Namibia and the south coast of South Africa [31]. The distribution of *M. paradoxus *overlaps with the region inhabited by *M. capensis *but this species is predominantly found along the west coast of South Africa [31]. Cape hake are migratory species that migrate seasonally and exhibit vertical migration [32]. These fish species are opportunistic predators and display a feeding pattern that is spatially and seasonally variable [32]. Cape hake were selected as model species for this study because these species are of high commercial interest around the world [33] and studies concerning the development and optimization of biomarker assays on *Merluccius *species are extremely limited [34].

## 2. Materials and Methods

### 2.1. Chemicals and Kits

Resorufin, 7-ethoxyresorufin, Coomassie Brilliant Blue R250, methanol, dl-isocitric acid trisodium salt, dicumarol, dl-dithiothreitol (DTT), bovine serum albumin (BSA), Bradford's reagent, and Ponceau S red and nitrocellulose membrane were supplied by Sigma-Aldrich, South Africa. Ethylenediamine tetraacetic acid disodium salt (EDTA), glycerol, glycine, sodium dodecyl sulphate (SDS), tris(hydroxymethyl)-aminomethane, Tween 20, methanol, magnesium sulphate, acetone, and 2-[4-(hydroxyethyl)-1-piperazinyl-ethanesulfonic acid (HEPES) were supplied by Merck, South Africa. Nicotinamide adenine dinucleotide phosphate (NADPH) was supplied by Calbiochem, South Africa. The BM chemiluminescence western blotting kit (mouse/rabbit) was supplied by Roche, South Africa, and rabbit anti-fish CYP1A peptide was supplied by Biosense Laboratories AS, Norway. 

### 2.2. Test Organism and Study Area

Frozen Cape hake samples were obtained from Sea and Coasts in Cape Town, South Africa and Balobi Trading, Mossel Bay, Eastern Cape, South Africa (*M. capensis *and *M. paradoxus, n* = 11). All Cape hake samples were transported at approximately −8 to −10°C and arrived frozen. Samples were thawed at 4°C overnight (on the day of arrival), and the total length (cm) and weight (g) were measured to determine Fulton's condition factors (CFs). Livers were excised and the weight noted (g). Excised samples were stored at −20°C until further analysis (thawed liver samples were retained for no longer than a month).

### 2.3. Preparation of Postmitochondrial and Microsomal Fractions

Liver preparations were carried out using a modified protocol of Nilsen [35]. All preparations were carried out at 4°C. Liver samples were thawed on ice, and preparation of the postmitochondrial fraction (PMS) was performed by homogenizing samples in 1 : 4 (w/v) cold homogenization buffer (10 mM HEPES, 1 mM DTT, 1 mM EDTA, 20% (v/v) glycerol) at pH 7.4 using a Waring commercial blender. Homogenates were subsequently centrifuged at 12,000 *×* g for 20 minutes in a Beckman Coulter J2-21 Avanti J-E centrifuge. The supernatant S1 sample was centrifuged at 40,000 *× *g for 2 hours to obtain the microsomal fraction (MS)/pellet 2 (P2), which was resuspended in 1 : 1 (w/v) resuspension buffer (50 mM Tris, 1 mM DTT, 1 mM EDTA, and 20% (v/v) glycerol), pH 7.4. PMS and MS fractions were retained and stored at −20°C. Protein concentrations for the two fractions were determined according to Bradford [36] at 595 nm using Bradford's reagent and bovine serum albumin as the protein standard. Sodium dodecylsulfate polyacrylamide gel electrophoresis (SDS-PAGE) analysis on the fractions was conducted and gels were stained with Coomassie Brilliant Blue dye, according to Laemmli [37]. 

### 2.4. Discontinuous 7-Ethoxyresorufin-*O*-Deethylase Activity (EROD) Spectrophotometric and Fluorescence Assays

EROD analysis was performed using a modified method described by Pikkarainen [38], on the PMS and MS fraction in Cape hake to determine which fraction contained the highest EROD activity. Comparative analysis using fluorimetry and spectrophotometry was performed to determine which detection technique yielded the most accurate results. NADPH (0.1 M) was added to a reaction mixture containing microsomal protein (10 *μ*L), 7-ethoxyresorufin (0.097 mg/mL in methanol), and tris-NaCl (TN) buffer (0.05 M Tris, pH 7.6, containing 0.1 M NaCl). The reaction mixture was incubated in a Labnet dry bath at 23°C for 15 minutes, after which time the reaction was terminated by the addition of 500 *μ*L ice cold methanol (99.5%). Samples were then centrifuged at 6,000 *× *g in a Heraeus Megafuge 1.0 R for 20 minutes at 4°C to remove any cellular debris that could interfere with the results. The resultant supernatant was analyzed spectrophotometrically at 572 nm [39, 40] in a Powerwave_X_ spectrophotometer using KC Junior software. Results were also analyzed using a Hitachi Spectrofluorometer F2500, using excitation and emission wavelengths of 510 and 585 nm, respectively [38]. All samples were prepared in triplicate with appropriate enzyme and substrate controls. Standard curves were constructed using commercial resorufin as a suitable standard.

### 2.5. Dot Blot Analysis of Microsomal Preparations

Dot blot analysis was used to determine the presence of CYP1A in different microsomal fractions that were obtained from differential centrifugation. A modified dot blot analysis was carried out as described by Desantis et al. [41]. The microsomal sample (2 *μ*L) was spotted onto a nitrocellulose membrane and incubated at 23°C until sample spots had completely dried. Immunoblot reactions (BioRad instructions) were carried out using primary (rabbit anti-fish CYP1A peptide) and secondary (anti-mouse/rabbit-antibody-horseradish peroxidase conjugate (POD)) antibody dilutions of 1 : 5000. The homogenization buffer was used as a negative control. No positive control was included as no commercial fish CYP1A for any fish species was available at the time of this study.

### 2.6. EROD Assay Optimization

EROD assay conditions were optimized for Cape hake with respect to pH (5.0, 6.0, 6.5, 7.0, 7.5, 8.0, and 8.5), reaction time (30 seconds to 40 minutes), temperature (23, 25, 30, 35, 40, and 45°C) and amount of enzyme (1, 2, 5, 10, and 15 *μ*L). The NADPH dependence of the EROD assay was also assessed by running parallel reactions in the absence and presence of the coenzyme (0.1 M). All experiments were conducted in triplicate with appropriate enzyme and substrate controls. Results were analyzed at 572 nm with a Powerwave_x_ spectrophotometer using KC Junior software.

### 2.7. Phase II Enzyme Inhibition Study

CYP1A was only partially purified from liver samples in Cape hake, and phase II metabolic enzymes that are present in solution are known to interfere with the EROD assay [42]. The effect of varying concentrations of MgSO_4_ (1, 2, 4, 6, 7, and 10 mM), isocitric acid (1, 2, 4, 6, 7, and 10 mM), and dicumarol (10, 20, 40, 60, 80, and 100 *μ*M) on the EROD assay was assessed (these compounds are reportedly all phase II metabolic enzyme inhibitors). Reactions were conducted in triplicate with appropriate enzyme and substrate controls. Results were analyzed at 572 nm as indicated above.

## 3. Results

**Figure 1:**
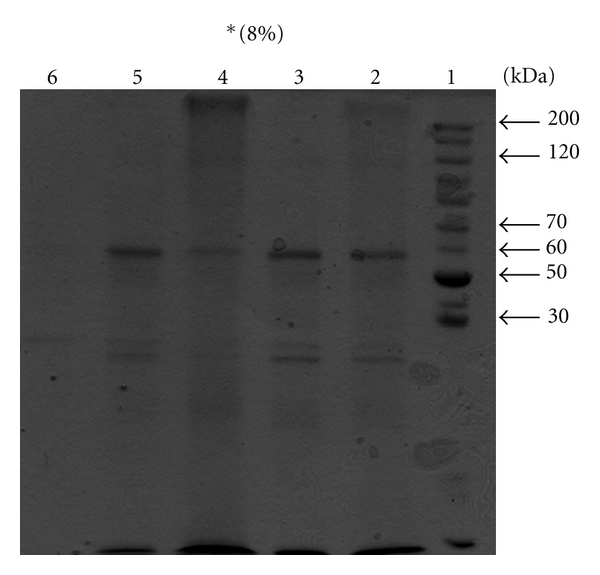
The SDS-PAGE profile of microsomal preparations of CYP1A in *M. capensis/M. paradoxus*. Lane 1: peqGOLD molecular weight marker; Lane 2: crude (4.800 mg/mL); lane 3: supernatant 1 (0.029 mg/mL); lane 4: pellet 1 (1.564 mg/mL); lane 5: supernatant 2 (2.721 mg/mL); lane 6: pellet 2 (0.014 mg/mL). *An 8% resolving gel was used.

**Figure 2:**
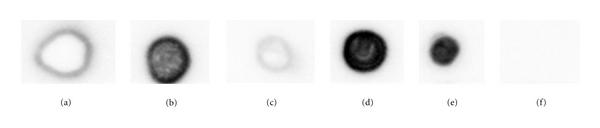
Dot blot analysis of CYP1A in different microsomal fractions in Cape hake. (a): Crude (9.600 *μ*g/*μ*L), (b): Supernatant 1 (0.058 *μ*g/2 *μ*L); (c): Pellet 1 (3.128 *μ*g/2 *μ*L); (d): Supernatant 2 (5.442 *μ*g/2 *μ*L); (e): Pellet 2 (0.028 *μ*g/2 *μ*L); (f) Negative control: Homogenization buffer. Two *μ*L of protein sample from each fraction were spotted onto the nitrocellulose membrane.

SDS-PAGE profiles of the liver fractions (Figure 1) obtained from Cape hake at different stages of the microsomal preparation procedure indicated the presence of three bands at approximately 60 kDa and two bands at ≤30 kDa in the crude fraction, PMS (supernatant 1), pellet 1 and supernatant 2. Two faint bands were also observed in the MS fraction (pellet 2) at molecular weights of approximately 60 kDa and below 30 kDa. The presence of CYP1A in the different fractions was confirmed by dot blot analysis (Figure 2), where fractions (a), (b), (c), (d), and (e) indicated positive immunoreactivity. A higher response signal was observed in fractions (d) (supernatant 2) and (e) (pellet 2), which led to the assumption that higher concentrations of CYP1A were present in these two fractions. 

**Figure 3:**
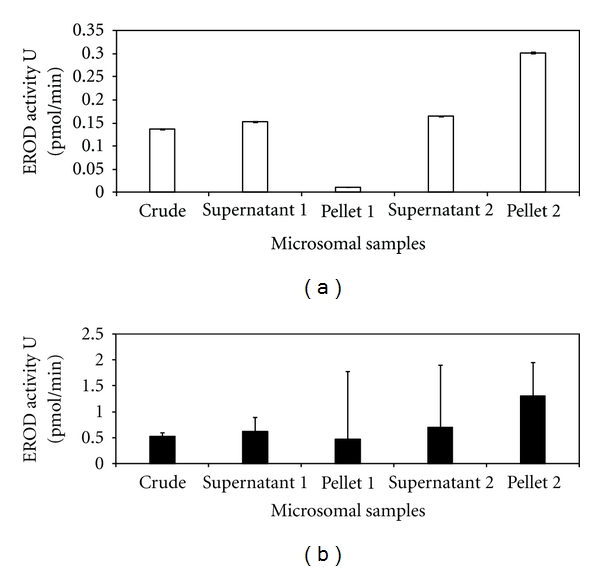
EROD activity (pmol/min) determinations for all fractions from the microsomal preparations in *M. capensis/M. paradoxus.* The study was conducted in triplicate using spectrophotometry (a) (wavelength: 572 nm) and fluorescence, (b) (excitation wavelength: 510 nm, emission wavelength: 58 nm). Data points represent mean values ± SD (*n* = 3).

The EROD assay was conducted on all fractions obtained from the microsomal preparations to establish which fraction displayed the highest EROD activity. Both spectrophotometric (Figure 3(a)) and fluorimetric (Figure 3(b)) results indicated that the highest EROD activity was present in pellet 2 (93 pmol/min/mg and 22 pmol/min/mg, resp.). The comparative study between fluorimetry and spectrophotometry indicated that there was a lower degree of variability (standard deviation) in the triplicate runs for the spectrophotometric data obtained. Spectrophotometric analysis was therefore selected as the most appropriate assay for the remainder of this study. 

**Figure 4:**
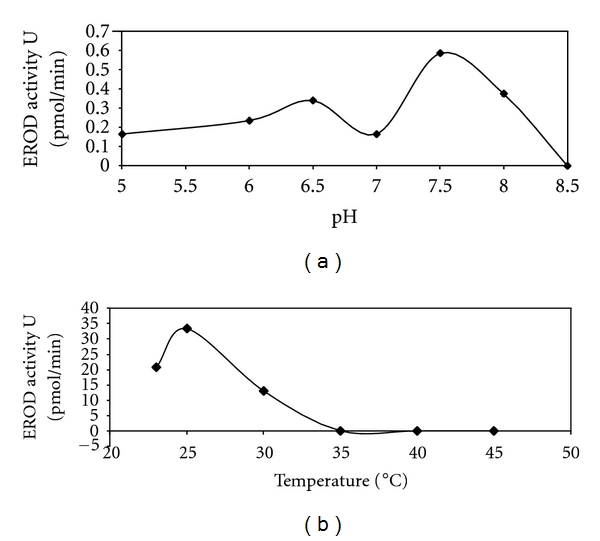
Assay optimization curves for *M. capensis/M. paradoxus *showing influence of pH (a) and temperature (b) on EROD activity in the pellet 2 fraction. Activities were determined spectrophotometrically at 572 nm and expressed as a % of maximal activity. Data points represent mean values ± SD (*n* = 3). Standard deviation bars are indicated but cannot be observed in cases were standard deviations were ≤0.0047.

**Figure 5:**
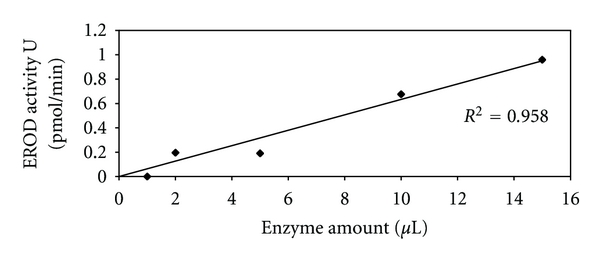
Assay optimization curves for *M. capensis/M. paradoxus *showing influence of enzyme amount on EROD activity in the pellet 2 fraction. Activities were determined spectrophotometrically at 572 nm and expressed as a % of maximal activity. Data points represent mean values ± SD (*n* = 3).

The EROD assay was optimized with respect to pH, temperature, reaction time, and enzyme volume for Cape hake samples (Figures 4 and 5). The EROD assay was observed to have a pH optimum of 7.5 (42 pmol/min/mg) (Figure 4(a)), although another smaller peak in activity was observed at pH 6.5 (24 pmol/min/mg). The optimum temperature for this reaction was observed at 25°C (2383 pmol/min/mg) (Figure 4(b)). The highest EROD activity was observed within 30 seconds (100%, 60 pmol/min; 4285 pmol/min/mg) of the assay (data not shown). A linear relationship was observed between EROD activity and increased amounts of enzyme (*r* = 0.958) (Figure 5), that is, an increase in the amount of enzyme amounts led to increased EROD activity. 

**Figure 6:**
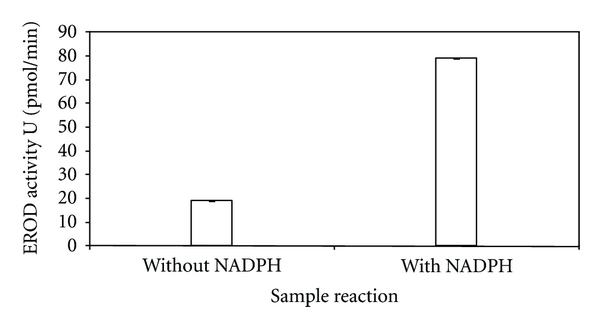
The NADPH dependence of the EROD activity in *M. capensis/M. paradoxus.* Results were obtained spectrophotometrically at 572 nm. Data points represent mean values ± SD (*n* = 3).

The NADPH dependence of the EROD assay (Figure 6) indicated that the EROD assay was limited by NADPH supply, as the addition of 0.1 M NADPH to the reaction showed a marked increase (300%) in EROD activity. Therefore, 0.1 M NADPH was added to all subsequent reactions for the duration of this study. 

**Figure 7:**
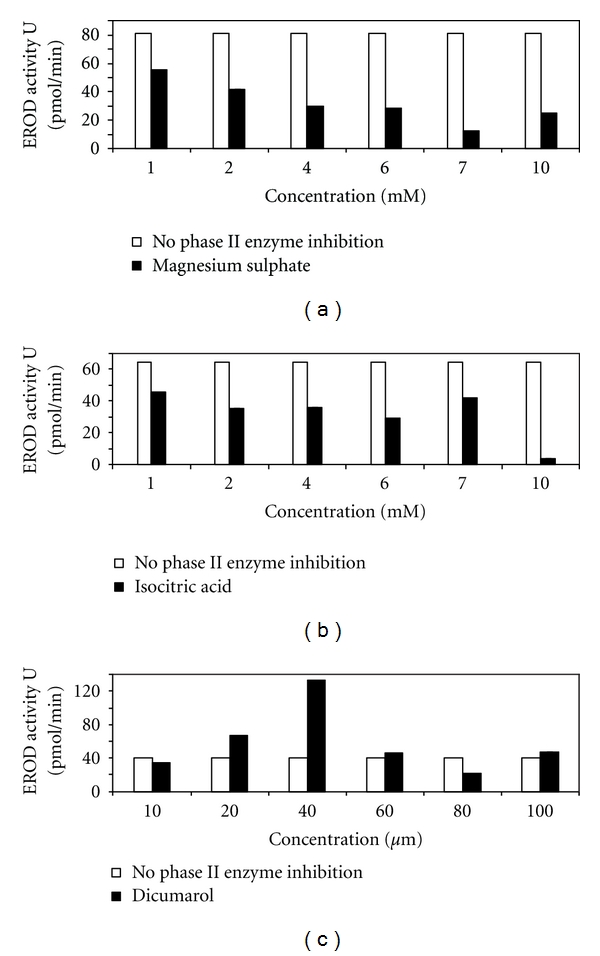
The phase II enzyme inhibition study in *M. capensis/M. paradoxus* using magnesium sulphate (a) isocitric acid (b), and dicumarol (c) as inhibitors. Results were obtained spectrophotometrically at 572 nm and expressed as a % of maximal activity. Data points represent mean values ± SD (*n* = 3). Standard deviation bars are indicated but cannot be observed in cases were standard deviations where ≤0.081.

 The effect of magnesium sulphate, isocitric acid, and dicumarol on phase II enzymes is demonstrated in Figures 7(a)–7(c). The addition of magnesium sulphate and isocitric acid (Figures 7(a) and 7(b)) was inhibitory to the EROD assay. Magnesium sulphate and isocitric acid were, therefore, not supplemented into EROD reactions for the remainder of the investigation. Increasing concentrations of dicumarol (Figure 7(c)) increased EROD activity with the highest activity being observed at a concentration of 40 *μ*M (100% activity, 133 pmol/min; 9488 pmol/min/mg). Dicumarol (40 *μ*M) was therefore added to EROD reaction mixtures for the duration of this study. 

## 4. Discussion

SDS-PAGE analysis performed on the fractions obtained from the microsomal preparation procedure (Figure 1) for the Cape hake samples indicated the presence of a major protein band at approximately 60 kDa and minor band(s) below 30 kDa in all fractions (crude, PMS-supernatant 1, pellet 1, supernatant 2, and MS-pellet 2). Although studies on CYP1A in *M. capensis/M. paradoxus *are limited, reports by Goksoyr and Forlin [43] have stated that fish CYP1A proteins have molecular weights ranging between 45 and 60 kDa (species dependent). Investigations by Mihailovic et al. [34] on the hake, *Merluccius merluccius, *reported that the molecular weight of CYP1A was 55 kDa. Most studies present the CYP1A protein as a monomer [25, 44]; therefore it was assumed that the lower molecular proteins observed in Figure 1 were degradation products formed during the isolation procedure. 

Dot blot analysis showed positive CYP1A immunoreactivity for all fractions including the PMS and MS fractions (Figure 2). High signal response was observed in supernatant 2 and pellet 2 which led to the assumption that these two fractions contained the highest concentrations of CYP1A (Figures 2(d) and 2(e)). This was confirmed by conducting the ethoxyresorufin-*O*-deethylase (EROD) assay on each fraction. EROD analysis results for the different fractions (Figures 3(a) and 3(b)) indicated that the overall EROD activity was the highest in pellet 2 (microsomal fraction), therefore this fraction was selected for all further analyses.

Both spectrophotometric and fluorimetric analyses were conducted in triplicate to test which method produced more accurate results. The fluorimetry results (Figure 3(b)) demonstrated greater variation within triplicate samples in EROD activities between runs. The feasibility of using these two detection methods has also been investigated by Klotz [39], although observations in their study are not consistent with results obtained for this study. In the Klotz [39] study fluorimetry was found to be slightly better than spectrophotometric analysis, in respect to sensitivity and accuracy. This study does, however, suggest that the spectrophotometric assay (Figure 3(a)) is a reliable method and has other advantages over the fluorimetric detection method; these include the visible assay being less laborious and the use of small assay volumes. Spectrophotometric analysis at 572 nm was therefore selected as the EROD assay detection method of choice for the duration of this study.

EROD assay conditions were optimized with respect to pH, temperature, enzyme volume, and time (Figures 4 and 5). The pH and temperature optima have been well documented in the literature for different fish species. Results observed for the time study (data not shown) reported the highest EROD activity after 30 seconds into the assay reaction. 

Addition of NADPH increased EROD activity by 300%, indicating that the EROD assay was limited by the amount of NADPH present in the assay. NADPH (0.1 M) was therefore added to all EROD reaction mixtures for the duration of this study. Although the EROD assay in this particular case was limited by exogenous NADPH supply, other studies using intact fish hepatocytes have demonstrated that the addition of this coenzyme to the overall EROD reaction is not required as the resulting increase in EROD activity was insignificant [45, 46]. The result observed in this study was not unexpected, as the centrifugation step separates the microsomal membranes from the NADPH present in the cytosol, whereas intact tissues have the biological capacity to generate endogenous NADPH [46].

A phase II enzyme inhibition study was performed to assess the degree of underestimation of EROD activity during this study. Considering that only partially pure CYP1A samples were analyzed, it is possible that phase II enzymes such as DT-diaphorase and other cytosolic oxidoreductases were present within the mixture. These enzymes may interfere with the EROD assay, as these enzymes have the capacity to further metabolize the product of the reaction, namely, resorufin [47]. The compounds magnesium sulphate, isocitric acid, and dicumarol have all been reported to inhibit these enzymes [42]. The addition of magnesium sulphate and isocitric acid had an overall inhibitory effect on the EROD assay, and they were thus not added into the reaction mixtures during this study (Figures 7(a) and 7(b)). The addition of 40 *μ*M dicumarol to the EROD reaction (Figure 7(c)) showed a significant increase in EROD activity from 40 pmol/min (in the absence of dicumarol) to 133 pmol/min (in the presence of dicumarol), which represented a 233% increase in overall activity. The effects of dicumarol on the EROD assay have been well established in the literature with similar findings being reported. Jönsson et al. [47] showed that the addition of 10^−5^ M of dicumarol increased resorufin concentrations by 33%. Das et al. [48] were in agreement with the findings of our study, and confirmed the reappearance of resorufin in an incubation system (by the addition of 10 *μ*M dicumarol) that was depleted of this product. Dicumarol was therefore included when performing EROD assays in the present study.

## 5. Conclusion

In summary, the results presented in this paper demonstrate the development and optimization of an EROD assay for Cape hake. Spectrophotometric analysis at (572 nm) was selected as the preferred detection method for this assay, as a lower degree of variability was obtained between data. Optimum conditions for the EROD assay were found to be as follows: pH of 7.5, temperature of 25°C, 10 *μ*L of enzyme, and a reaction time of 30 seconds. The EROD assay was, however, limited by NADPH supply. The addition of the phase II inhibitor dicumarol significantly increased EROD activity. In conclusion, the EROD assay conditions for Cape hake were optimized in order to provide a method for future studies involving the detection of this pollution biomarker in Cape hake and related species of fish. A few recommendations should be considered for future studies assessing the effect of environmental exposure. All Cape hake samples used in this study arrived frozen which is suitable for this assay optimization study. However, future studies should consider on-site sample preparation (liver excision and nitrogen fixing) as this has been proven to reduce the loss of enzyme activity and variation in individual activity, which could be the result of freezing and thawing of whole fish samples. Future studies could also employ the use of liver samples obtained from fish exposed to AhR agonists as positive controls, in the event fish CYP1A cannot be obtained commercially.
